# High-Performance Quasi-Two-Dimensional CsPbBr_2.1_Cl_0.9_:PEABr Perovskite Sky-Blue LEDs with an Interface Modification Layer

**DOI:** 10.1186/s11671-022-03703-6

**Published:** 2022-07-22

**Authors:** Yi-Tsung Chang, Lingun Zhang, Mu-Jen Lai, Wei-Chen Chiang, Lung-Chien Chen

**Affiliations:** 1 Department of Physics, School of Science, Jimei University, Xiamen, 361021 China; 2 Jiangxi Litkconn Academy of Optical Research Co., Ltd, Longnan City, 341700 Jiangxi China; 3 Department of Electro-Optical Engineering, National Taipei University of Technology, Taipei, Taiwan

**Keywords:** Interface modification, Electron transport layer, Quasi-two-dimensional Perovskites, PEABr, CsPbBr_2.1_Cl_0.9_, Sky-blue LED

## Abstract

This paper elucidates the increased luminescence efficiency of CsPbBr_2.1_Cl_0.9_ sky-blue perovskite light-emitting diodes (PeLEDs) achieved through the interface modification of 3,4 ethylenedioxythiophene (PEDOT):polystyrene sulfonic acid (PSS)/quasi-two-dimensional (QTD) perovskite using CsCl and CsBr materials, respectively. QTD films were fabricated using ratios of CsPbBr_2.1_Cl_0.9_ doped with phenethylamine hydrobromide (PEABr) at 60%, 80%, and 100%. The solvent dimethyl sulfide (C_2_H_6_OS) was employed under the excitation of ambient and 365-nm laser lights. The PeLED structure was composed of Al/LiF/2,2',2"-(1,3,5-benzinetriyl)-tris(1-phenyl-1-H-benzimidazole) (TPBi)/CsPbBr_2.1_Cl_0.9_:PEABr/interface modification layer/PEDOT:PSS/ITO glass. The optimized results revealed that the luminance, current efficiency, and external quantum efficiency of the QTD CsPbBr_2.1_Cl_0.9_:80% PEABr PeLED with the CsCl interface modification additive was 892 cd/m^2^, 3.87 cd/A, and 5.56%, respectively.

## Introduction

In 1839, the Russian mineralogist von Perovski discovered perovskite [1]. Halide perovskite materials were to play a critical role in the history of industrial evolution and were successfully applied in the field of solar cells [2, 3], light-emitting diodes (LEDs) [4, 5], lasers [6, 7], and photodetectors [8, 9]. Perovskite has material characteristics that vary depending on the zero-dimensional to three-dimensional (3D) structures employed. Its favorable properties include its adjustable energy band for light emission, wide color gamut, and low cost. Perovskite can convert electrical energy into light energy, which can be applied to LEDs; thus, perovskite light-emitting diodes (PeLEDs) are widely applied. Significant development has been achieved in green and red light PeLEDs, with their respective illuminance reaching approximately 500,000 and 20,000 cd/m^2^ [10]. Comparatively, the development of blue PeLEDs has been slow [11–14]. Perovskite materials applied in quantum dots (QDs) and zero-dimensional and 3D structures are easily affected and decomposed by moisture and oxygen in the environment, which indirectly affects the light emission efficiency of the devices. Hence, effective perovskite devices are difficult to fabricate. Scientists have therefore developed a type of perovskite through the introduction of large organic cations as spacers to form a quasi-two-dimensional (QTD) perovskite and have applied it to full-color display and white light illumination [15–22].

Numerous studies have analyzed perovskite and its application since its discovery in 1831. For example, in 2016, Cheng et al. discovered that adding numerous organic cations to all-inorganic perovskites can form a QTD layered Ruddlesden-Popper (R-P; V(RNH_3_)_2_A_*n*-1_B_*n*_X_3*n*+1_) crystal phase. Organic cations can effectively form a layered structure with high film coverage and luminescence performance, which can be used to prepare high-efficiency sky-blue PeLED with low starting voltage and high external quantum efficiency (EQE) [23]. In 2018, Hou et al. used a manganese (Mn) ratio to replace the lead (Pb) content in perovskite QDs. The results demonstrated that the appropriate addition of manganese can effectively increase the quantum yield and photoinduced luminous production rate and lifetime, reduce trap density, and achieve a illuminance and EQE of 389 cd/m^2^ and 2.12%, respectively [24]. In 2019, Sun et al. published a synthesis method based on zero-dimensional and 3D perovskite anion exchange. Through a simple anion exchange reaction, the quantum yield of perovskite can be enhanced to 90% using surface reconstruction technology. A method for improving the quantum yield, which used blue PeLEDs with a wavelength of 463 nm combined with other blue perovskites as a novel type of intelligent invisible ink for information encryption and decryption, has considerable potential for anticounterfeiting and information security applications [25]. In 2019, Jin et al. introduced two organic long-chain ligands (PEABr and NPABr), and the QTD perovskite formed with these dual ligands is highly stable. The blue film achieved the highest EQE of 2.62%, and the highest illuminance was 1200 cd/m^2^ at 5.8 V [26]. Li et al. developed a PEDOT:PSS/QTD perovskite crystal with vertical nonuniform phase distribution in 2019. Deionized water was used to dilute the consistency of PEDOT:PSS. The thickness of the spin-coated hole injection layer was adjusted to the position of the recombination zone. The highest EQE and illuminance achieved were 5.7% and 3780 cd/m^2^, respectively [27]. In 2020, Wang et al. applied double-long-chain ligands to prepare QTD perovskite films with appropriate proportions of phenethylammonium (PEA) and isobutylammonium. The ligands can effectively enhance photoluminescence (PL) performance, uniformity, and intensity. Through adjustment of the ratio of the two ligands, a sky-blue PeLED with an electroluminescence (EL) peak at 485 nm can be achieved, with illuminance of 1130 cd/m^2^ and EQE as high as 7.84% [28]. In 2021, Yan et al. used zinc (Zn) to partially replace the lead (Pb) content. ZnBr_2_ could effectively solve the problems of ion defects during filling and size–structure characteristic conversion. The binding force of the ore changed from van der Waals forces with PEA to ionized van der Waals forces with ZnBr_2_, achieving a maximum illuminance and EQE of 1245 cd/m^2^ and 1.3%, respectively [29]. In 2018, Yang et al. reported a QTD green light perovskite with a R–P crystal phase that exhibited increased exciton binding energy and fewer charge carriers. Converting a 3D perovskite into a QTD perovskite introduces many defects on the surface or at the grain boundary. To reduce the surface defects, the surface modification process in a previous study involved coating the surface of the QTD perovskite film with organic small-molecule trioctylphosphine oxide; the current efficiency reached 62.4 cd/A, and the EQE was 14.36% [30]. In 2019, Hoye et al. used perovskite nanoplatelets to prepare a nanoplatelet PeLED for the hole layer. Severe nonradiation recombination occurred in the perovskite nanosheet layer, which led to the overall efficiency of the device [31]. The additional introduction of poly(triarylamine) as an intermediate layer effectively reduced the nonradiation recombination, and the measured maximum EQE of the PeLED chip under pure blue light (464 nm) increased from 0.007 to 0.3%. In 2019, Tang et al. applied 2,2'-(ethylenedioxy)bis(ethylamine) (EDBE) to modify the ZnMgO electron transport layer (ETL) and perovskite light-emitting layer. EDBE has two amino groups. One amine can interact with ZnMgO below to improve the growth and electron injection of the perovskite film, effectively reducing the leakage current. The other amine can modify the trap state on the surface to effectively reduce nonradiation. After the reorganization, the NIR perovskite LED element with and without the addition of a modified layer was compared. The maximum EQE increased from 9.15 to 12.35% [32]. In 2020, Wang et al. used rubidium chloride (RbCl) to modify the interface between PEDOT:PSS and the perovskite film, which effectively improved blue light. The PL and EL properties of the 3D perovskite film achieved a maximum illuminance and EQE of 9243 cd/m^2^ and 1.66%, respectively [33]. In 2021, Shen et al. employed a heterostructure using potassium cations (K^+^) to coat the hole injection layer (PEDOT:PSS), enhancing the hydrophilicity of the perovskite. As a result of the ionic interaction between K^+^ and the halogen anion (Br^−^ or Cl^−^), the recombination probability and hole transport ability increased, and a maximum EQE and illuminance of 4.14% and 451 cd/m^2^, respectively, were achieved at an EL wavelength of 469 nm [34]. Interface modification is applied to reduce the defects generated between the film layers but must be tailored to the various forms of perovskite, including 3D nanofilms, two-dimensional (2D) nanosheets, one-dimensional nanowires, and zero-dimensional QDs. Perovskite structures, from 3D to zero-dimensional structures, can be modulated through different deployment methods. In 2021, Mohammed et al. fabricated perovskite solar cells (PSCs) based on low-temperature solution-processed ZnO nanoparticles and the thermal stability of PSCs based on the ZnO/CNT ETL in ambient has boosted [35]. Also, a facile and effective doping engineering approach based on graphene quantum dots (GQDs) was introduced to modify the SnO_2_/ZnO bilayer electron transport layer (ETL). The SnO_2_/ZnO layers with altered concentrations of GQDs improved significantly the properties and reliability [36]. Besides, Hussein et al. addressed mixed cation of cesium (Cs)/formamidinium (FA)/methylammonium (MA) for PSCs via an air-processed sequential deposition method [37]. In 2022, Kareem et al. employed 3D/2D bi-layer to improve the properties of FAPbI_3_-based PSCs and promoted intrinsic and extrinsic stability issues of PSCs [38]. Besides, in visible blue light, sky blue has lower energy, help to reduce glare, and improve contrast. Therefore, in this study, PEABr was prepared to form a QTD sky blue PeLED, and interface modification layers of CsCl and CsBr were added into the QTD sky blue PeLED structure, which was further investigated and analyzed.

## Experimental Procedure

### Materials and Precursors

The chemicals used to fabricate PeLEDs in this study were cesium bromide (CsBr), cesium chloride (CsCl), phenethylamine hydrobromide (PEABr), lead(II) bromide (PbBr_2_), 2,2',2"-(1,3,5-benzinetriyl)-tris(1-phenyl-1-H-benzimidazole) (TPBi), dimethyl sulfoxide (DMSO), ethanol acetone, and 3,4 ethylenedioxythiophene (PEDOT):polystyrene sulfonic acid (PSS).

The CsPbBr_2.1_Cl_0.9_:PEABr perovskite source materials used in this study were CsCl, CsBr, PbBr_2_, and PEABr. The preparation procedure is detailed as follows: A precursor solution of CsPbCl_x_Br_3− x_:PEABr was prepared. The 1:1 ratio of Pb and Cs was maintained, and CsCl (0.18 mmol), CsBr (0.02 mmol), and PbBr_2_ (0.2 mmol) were added; an appropriate amount of PEABr was dissolved in 1 mL of DMSO. Then, the solution was placed on a rotary table and stirred for 12 h to achieve complete dissolution of the 2D perovskite solution. The interface modification layers in this study employed CsCl and CsBr. The mixed solution at a ratio of 4 mg:1 mL of CsCl (or CsBr) and DMSO was placed in the glove box of a heating rotary table at 600 rpm at 25 °C. The solution was then placed on the rotary table and stirred for 12 h to reach a completely dissolved state.

### Device Process

**Fig. 1:**
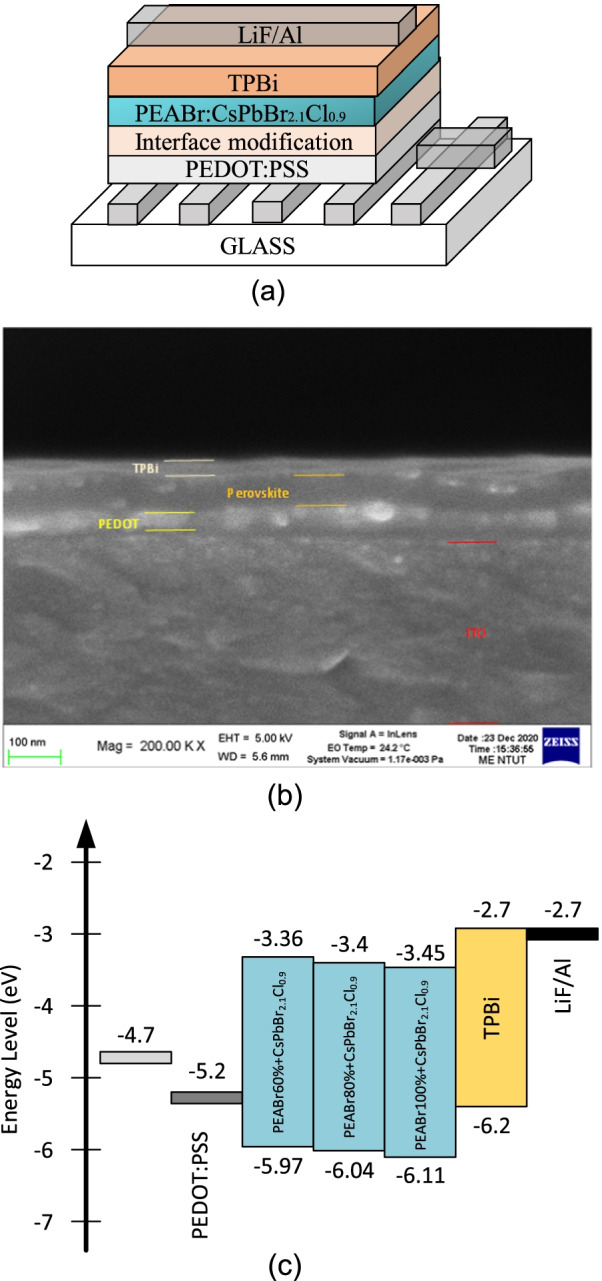
(**a**) Structure, (**b**) SEM image, and (**c**) electron level of the QTD CsPbBr_2.1_Cl_0.9_:PEA PeLED

First, a clean and prepared ITO glass substrate with an area of 1.5 × 1.5 cm was placed into an ultraviolet-ozone cleaner (UV-Ozone Cleaner) and irradiated for 20 min, which can effectively improve the cleanliness of the surface and enhance the hydrophilicity of the ITO substrate surface. The PEDOT:PSS hole injection layer and the CsCl (or CsBr) interface modification layer were spin-coated on the ITO glass substrate at 4500 rpm for 45 s. After spin coating, the substrate was placed on a heating plate and baked at 150 °C for 15 min. Next, the completely dissolved QTD perovskite CsPbBr_2.1_Cl_0.9_:PEABr solution was spin-coated on the CsCl (or CsBr)/PEDOT:PSS/ITO substrate at 3500 rpm for 50 s in the glove box. The substrate was then transferred to the hot plate and baked at 70 °C for 5 min. Subsequently, a 30-nm-thick TPBi ETL was deposited on the QTD perovskite CsPbBr_2.1_Cl_0.9_:PEABr active layer using a thermal evaporator. The thermal evaporator was pumped down to a high vacuum environment (approximately 2 × 10^−6^ torr) using a hydraulic diffusion pump. The film deposition rate was 1 Å/s. Finally, 1-nm-thick LiF and 100-nm-thick Al were sequentially deposited on the TPBi ETL as cathode electrodes using the thermal evaporator. The PeLED structure, SEM image, and electron level are depicted in Fig. 1.

## Results and Discussion

**Fig. 2:**
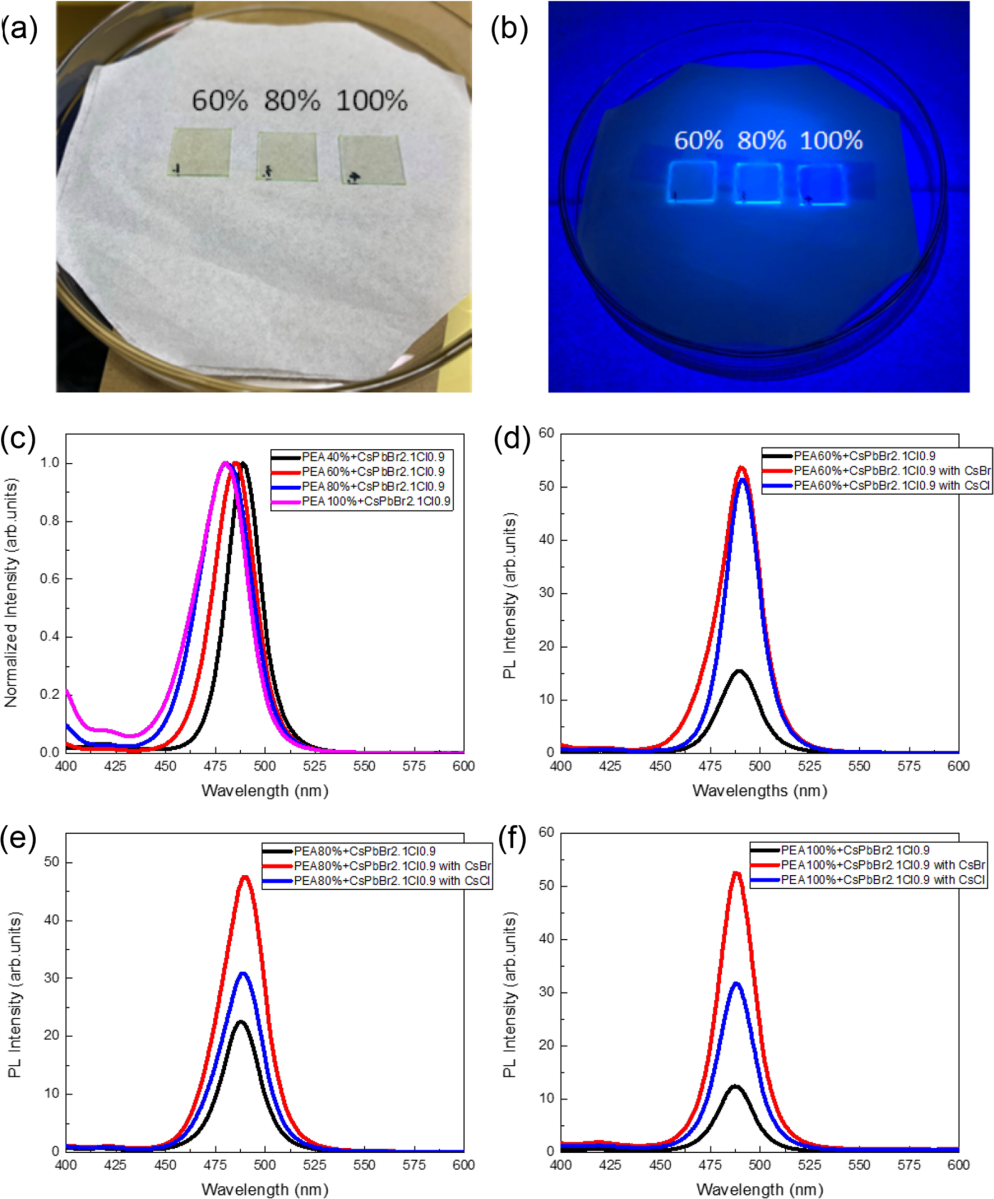
The CsPbBr_2.1_Cl_0.9_:PEABr film (**a**) without and (**b**) with excitation. (**c**) PL spectra of the CsPbBr_2.1_Cl_0.9_:x% PEABr film without an interface modification layer. PL spectra of the CsPbBr_2.1_Cl_0.9_:x% PEABr film with an interface modification layer, where x = (**a**) 40%, (**b**) 60%, (**c**) 80%, and (**f**) 100%

To verify the successful synthesis of the CsPbBr_2.1_Cl_0.9_:x% PEABr film (x = 60%, 80%, and 100%), the developed films were examined under laser excitation with a wavelength of 365 nm. Figures 2a and b present the CsPbBr_2.1_Cl_0.9_:PEABr film with and without excitation.

The PL spectra of the CsPbBr_2.1_Cl_0.9_:x% PEABr film (x = 40%, 60%, 80%, and 100%) and ratios of CsPbBr_2.1_Cl_0.9_ and PEABr (1:0.6, 1:0.8, and 1:1) are illustrated in Fig. 2c. The detailed parameters are listed in Table 1. When the proportion of PEABr was increased from 40 to 80% and 100%, the excited peak exhibited the blueshift phenomenon, because the 2D PEABr emission wavelength was close to 480 nm [12]. The CsCl and CsBr interface modification layers are depicted in Fig. 2d–f, and the detailed data are presented in Table 2. The PL peaks of the CsPbBr_2.1_Cl_0.9_:PEABr layers with and without the interface modification layer were almost the same, although the intensity of the PL spectra of the CsPbBr_2.1_Cl_0.9_:PEABr layers with the interface modification layer increased, as illustrated in Fig. 2c and d. Thus, an interface modification layer can effectively improve the intensity of PL spectra owing to surface defect decrease resulting from the addition of CsCl or CsBr, and the surface passivation effect of CsBr is better than that of CsCl. For the PL spectra, the peak position shifts and FWHM widen after adding CsBr and CsCl. It may be contributed from the both of CsPbBr_2.1_Cl_0.9_ and PEABr monolayer phase.

**Table 1:** PL peak and FWHM data of the QTD perovskite with different PEABr mixing ratios (40%, 60%, 80%, and 100%)

Ratio	40%PEABr	60%PEABr	80%PEABr	100%PEABr
Peak (nm)	488.8	484.8	480.6	479.2
FWHM (nm)	19.86	23.84	29.38	27.10

**Table 2:** PL peak and FWHM data of the QTD perovskite/interface modification layer/PEDOT/ITO structures with different PEABr mixing ratios (60%, 80%, and 100%)

Samples	60% PEABr	60% PEABr with CsCl	60% PEABr with CsBr	80% PEABr	80% PEABr with CsCl	80% PEABr with CsBr	100% PEABr	100% PEABr with CsCl	100% PEABr with CsBr
Peak (nm)	489.4	490	490.6	488	488.6	489	487.4	487.6	488
FWHM (nm)	22.61	23.31	20.15	21.73	24.75	25.61	20.87	21.35	21.25

**Fig. 3:**
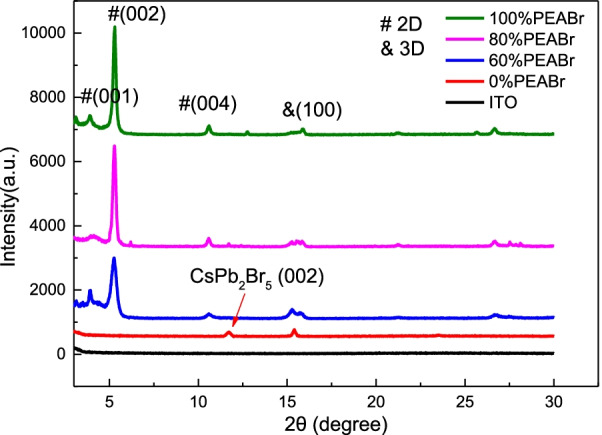
XRD patterns of the QTD perovskites with various proportions of PEABr

The XRD patterns of the QTD CsPbBr_2.1_Cl_0.9_:x% PEABr film (x = 60%, 80%, and 100%) are presented in Fig. 3. The XRD pattern indicated that the 3D perovskite without PEABr had two obvious diffraction peak: one position is at 15.4° corresponding to (100) plane of the CsPbBr_2.1_Cl_0.9_; another one is at 11.6°, corresponding to (002) plane of the CsPb_2_Br_5_, respectively. The phase of CsPb_2_Br_5_ in the inorganic halide perovskite is very easy to form as PEABr is not contained in the precursor. The relative peaks within the structure and the characteristic peaks of the QTD perovskite CsPbBr_2.1_Cl_0.9_ doped with PEABr at approximately 3.9°, 5.3°, and 10.6° (corresponding to 2D perovskite (001), (002), and (004) planes, respectively) were attributed to the cubic crystal structure; when the proportion of doped PEABr increased, the relative intensity of the diffraction peaks at 3.9° and 5.3° also increased [39, 40]. Thus, when the proportion of doping increased, the 3D perovskite structure transformed into a QTD perovskite structure. Furthermore, a representative 3D perovskite structure was observed near the 15.4° diffraction peak. This demonstrated that 2D and 3D perovskite structures were successful fabricated in this study and combined to form a QTD perovskite structure.

**Fig. 4:**
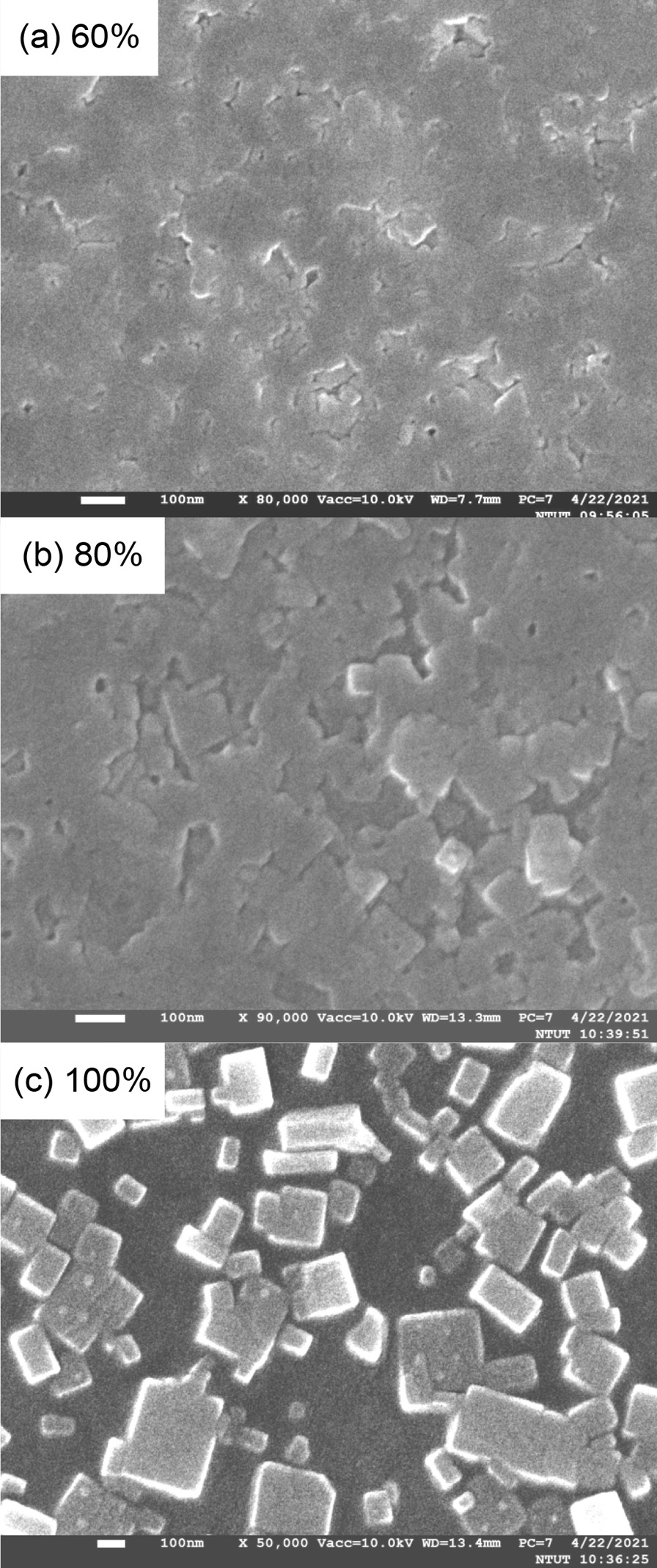
Top-view SEM images of the QTD CsPbBr_2.1_Cl_0.9_:PEABr films with varying proportions of PEABr (60%, 80%, and 100%)

Figure 4 depicts the top-view SEM images of the QTD CsPbBr_2.1_Cl_0.9_:x% PEABr films with varying proportions of PEABr (60%, 80%, and 100%). When 60% PEABr was doped in perovskite, the surface quality of the film was unfavorable, exhibiting numerous voids; additionally, perovskite crystallinity was not obvious. Under doping with 80% PEABr, some perovskite crystallinity was detected, with the crystal size of perovskite becoming distinct under 100% PEABr. Generally, the higher the proportion of PEABr added to the perovskite solution, the more improved the morphology and coverage, producing high-quality perovskite film, especially under 100% PEABr doping.

**Fig. 5:**
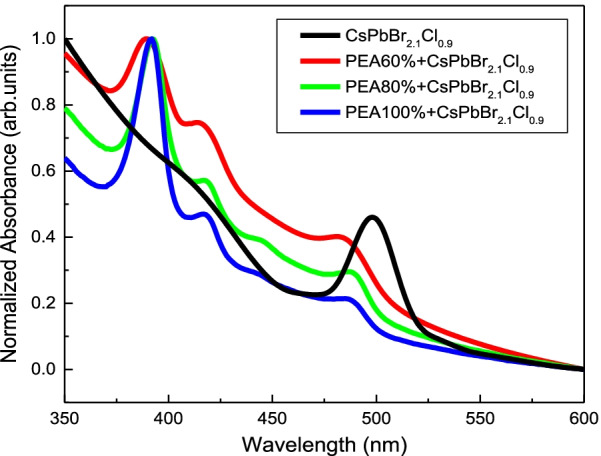
Absorbance spectra of the QTD CsPbBr_2.1_Cl_0.9_:PEABr films with varying proportions of PEABr (0%, 60%, 80%, and 100%)

Figure 5 presents the absorbance spectra of the QTD CsPbBr_2.1_Cl_0.9_:x% PEABr films with 0%, 60%, 80%, and 100% PEABr, respectively. The perovskite CsPbBr_2.1_Cl_0.9_ film exhibited bulk perovskite property under no PEABr (0%), whereas the CsPbBr_2.1_Cl_0.9_:x% PEABr films (x = 60%, 80%, and 100%) exhibited the R-P QTD perovskite property. The peak positions in the absorption spectra were at approximately 390, 420, 450, and 480 nm corresponding to one, two, three, and four monolayers phase [12]; the bandgap of the QTD CsPbBr_2.1_Cl_0.9_:PEABr corresponding to the number of monolayers was 3.20, 2.95, 2.76, and 2.58 eV. This result was obtained that it is owing to the exciton absorption of the CsPbBr_2.1_Cl_0.9_:PEABr film increased when the proportion of PEABr increased in the single monolayer phase.

**Fig. 6:**
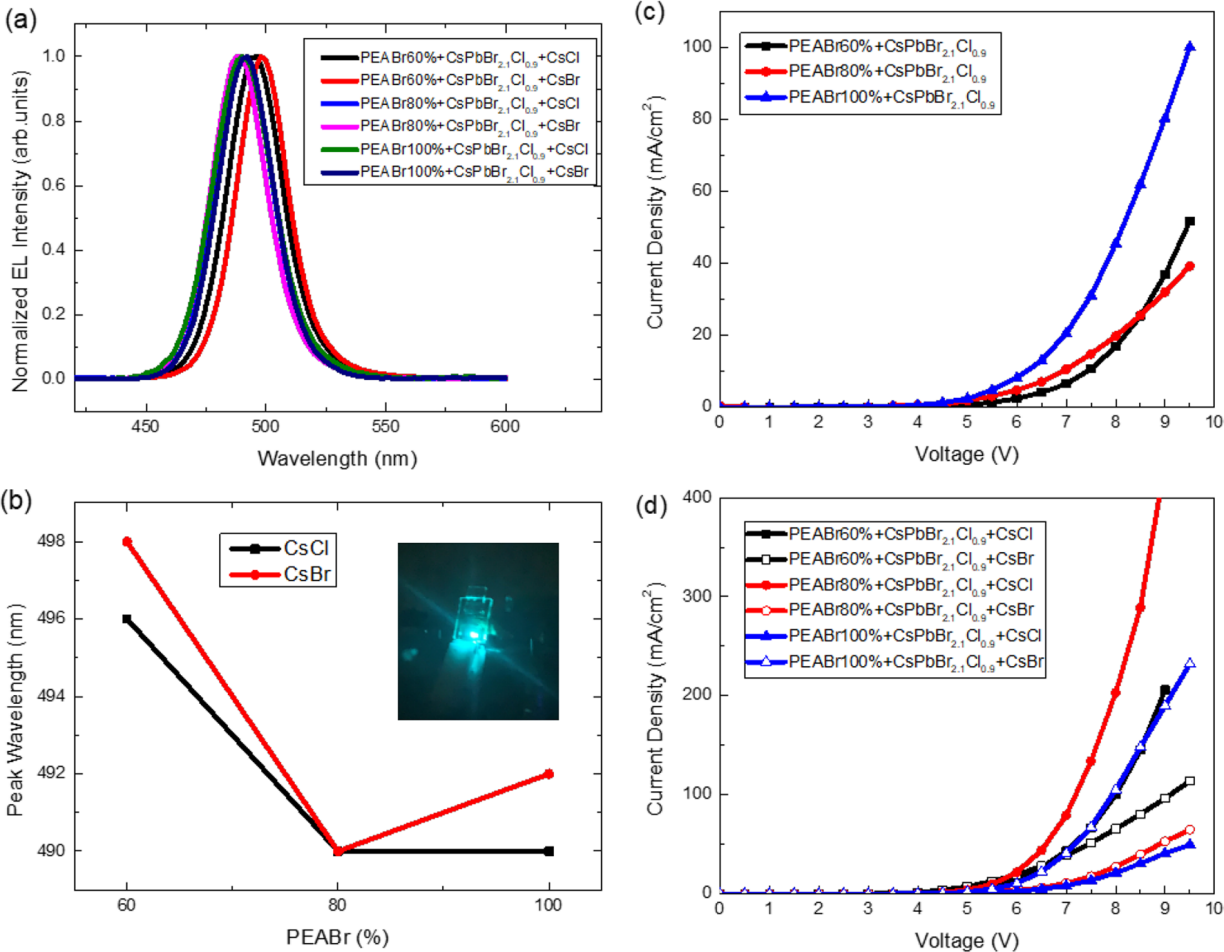
(**a**) Electroluminescence (EL) spectra and (**b**) EL peaks of the QTD CsPbBr_2.1_Cl_0.9_:PEABr PeLEDs with different additives (insert: the operational CsPbBr_2.1_Cl_0.9_:80% PEABr PeLED with CsCl additive). Current density of the QTD CsPbBr_2.1_Cl_0.9_:PEABr PeLEDs (**c**) without and (**d**) with different additives

**Fig. 7:**
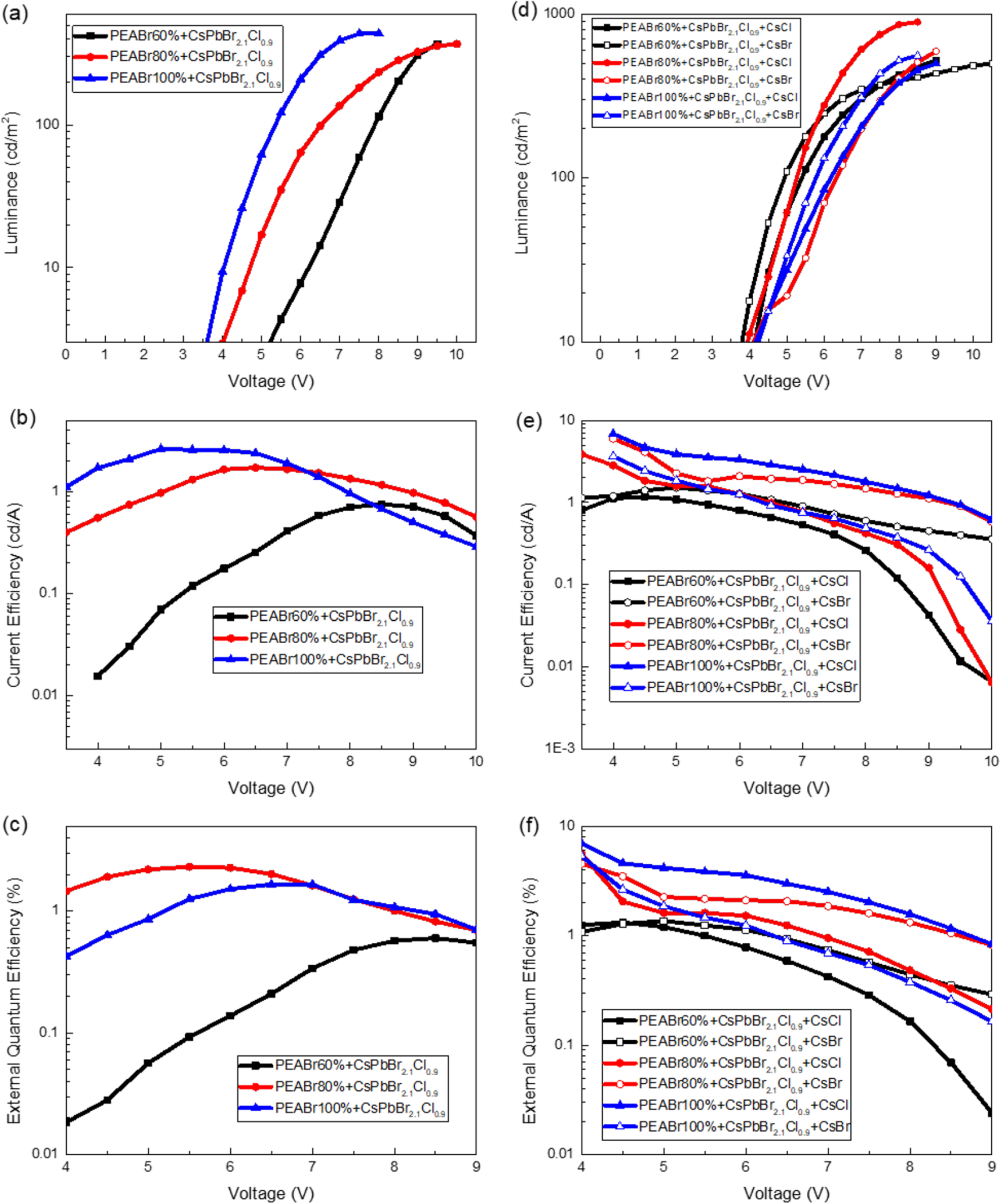
(**a**) Luminance, (**b**) current efficiency, and (**c**) external quantum efficiency of the QTD CsPbBr_2.1_Cl_0.9_:PEABr PeLEDs without different additives. (**d**) Luminance, (**e**) current efficiency, and (**f**) external quantum efficiency of the QTD CsPbBr_2.1_Cl_0.9_:PEABr PeLEDs with different additives

**Fig. 8:**
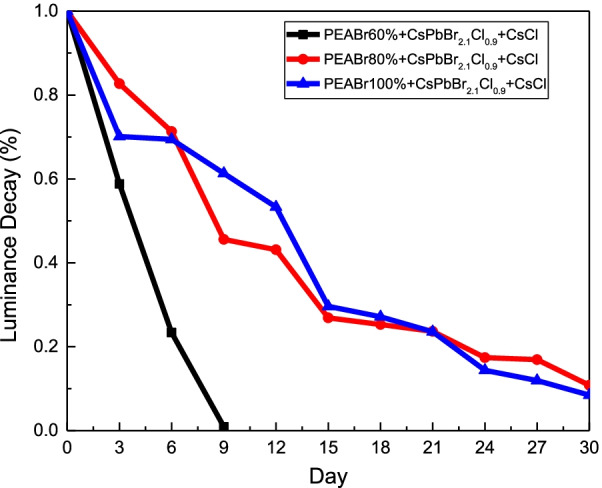
Reliability results of the QTD CsPbBr_2.1_Cl_0.9_:PEABr PeLEDs with CsCl interface modification additive

Figures 6a and b illustrate the plotting of the EL spectra and EL peak wavelengths of the QTD CsPbBr_2.1_Cl_0.9_:PEABr PeLEDs with CsCl or CsBr additives. The inset in Fig. 6b depicts the operating CsPbBr_2.1_Cl_0.9_:80% PEABr PeLED with the CsCl additive. The active area of the PeLED was 4 mm^2^, and compared with the PL spectra, the peaks of the EL spectra exhibited a redshift. This phenomenon occurred in the EL spectra owing to the applied electrical field. The EL peak position of the CsPbBr_2.1_Cl_0.9_:80% PEABr PeLED with the CsCl additive was slightly lower than that of the CsPbBr_2.1_Cl_0.9_:80% PEABr PeLED with the CsBr additive, which may have resulted from the wider energy gap of CsPbCl_3_ (2.98 eV) than that of CsPbBr_3_ (2.4 eV). The turn-on voltage was 4 V, and the current density substantially improved, as illustrated in Fig. 6c and d, through the reduced series resistance resulting from the modification of defects on the CsPbBr_2.1_Cl_0.9_:PEABr films. The turn-on voltage did not change with the introduction of the CsCl and CsBr interface modification layers. Figure 7 presents the illuminance, current efficiency, and EQE of the QTD CsPbBr_2.1_Cl_0.9_:PEABr PeLEDs without and with the additives. As depicted in Fig. 7a–c, the illuminance, current efficiency, and EQE of the QTD CsPbBr_2.1_Cl_0.9_:PEABr PeLEDs without an interface modification additive were approximately 400 cd/m^2^, 1 cd/A, and 1%, respectively; with an interface modification additive, these values increased significantly, as illustrated in Fig. 7d–f. Thus, the addition of either of these interface modification layers can effectively improve current efficiency. The surface passivation and interface modification of the CsCl and CsBr additives may have contributed to this improvement. The parameters of all the QTD perovskite PeLEDs are listed in Table 3 for comparison. The highest illuminance, current efficiency, and EQE, namely 892 cd/m^2^, 3.87 cd/A, and 5.56%, respectively, were achieved with the QTD CsPbBr_2.1_Cl_0.9_:80% PEABr PeLED with the CsCl interface modification additive. Figure 8 presents the reliability results of the QTD CsPbBr_2.1_Cl_0.9_:PEABr PeLEDs with the CsCl interface modification additive at a bias voltage of 6 V under an atmospheric environment. The decay ratio of the CsPbBr_2.1_Cl_0.9_:60% PEABr PeLEDs with the CsCl interface modification layer was more severely quenched, with the device exhausted by the ninth day. By contrast, the CsPbBr_2.1_Cl_0.9_:PEABr PeLEDs with the CsCl interface modification layer remained operational after 30 days.

**Table 3:** Turn-on voltage (V_on_), illuminance (L), current efficiency (CE), and external quantum efficiency (EQE) of the PeLED structures

Samples	60% PEABr	60% PEABr with CsCl	60% PEABr with CsBr	80% PEABr	80% PEABr with CsCl	80% PEABr with CsBr	100% PEABr	100% PEABr with CsCl	100% PEABr with CsBr
V_on_ (V)	4	4	4	4	4	4	4	4	4
L (cd/m^2^)	369	523	496	312	892	592	436	498	556
CE (cd/A)	0.75	1.12	1.52	1.81	3.87	5.99	2.62	6.84	3.65
EQE (%)	0.6	1.32	1.36	1.67	5.56	4.53	2.28	6.92	5.31

## Conclusions

In summary, high-performance QTD perovskite CsPbBr_2.1_Cl_0.9_:PEABr films were studied in terms of optimized material and optoelectronics properties. In this study, 2D PEABr was introduced into 3D perovskite, effectively transforming it into a QTD perovskite film. Furthermore, two interface modification layers, CsCl and CsBr, were compared, and the performance of the CsPbBr_2.1_Cl_0.9_:PEABr PeLED with the CsCl interface modification additive was determined to be the most effective. The CsPbBr_2.1_Cl_0.9_:100% PEABr PeLED with CsCl exhibited the highest EQE and current efficiency of 6.92% and 6.84 cd/A, respectively. The CsPbBr_2.1_Cl_0.9_:80% PEABr PeLED with the CsCl interface modification additive exhibited the highest illuminance of 892 cd/m^2^. Thus, the introduction of an interface modification layer can reduce defects on the surface and improve the performance of PeLEDs through effective electron–hole recombination in the active layer. Finally, the 2D PEABr can improve the stability and reliability of PeLEDs according to the results of the aging test.

## Data Availability

All the data are fully available without restrictions.

## Abbreviations

QTD: Quasi-two-dimensional
CsPbBr_2.1_Cl_0.9_: Cesium lead bromide chloride
PEABr: Cesium bromide
PbBr_2_: Lead bromide
DMSO: Dimethyl sulfoxide
PEABr: Phenethylamine hydrobromide
EQE: External quantum efficiency
FESEM: Field-emission scanning electron microscopy
ITO: Indium tin oxide
PEDOT:PSS: Poly (3,4-ethylenedioxythiophene) polystyrene sulfonate
PeLED: Perovskite light-emitting diode
